# Evaluating the Impact of Inspiratory Muscle Training on Respiratory Function and Exercise Capacity in Pulmonary Hypertension: A Systematic Review and Meta-Analysis of Randomized Controlled Trials

**DOI:** 10.3390/arm94010013

**Published:** 2026-02-15

**Authors:** Saja Alrashedi, Lama Alharbi, Meshal Alotaibi, Inad Alzahrani, Albara Jad, Qamar Aldoboke, Suroor Algethami, Raghda Alrabah, Rana Alharbi, Ali Al Nuwaiser, Mohammed Al-Hariri

**Affiliations:** 1 Department of Physical Therapy and Health Rehabilitation, College of Applied Medical Sciences, Jouf University, Sakaka 72388, Saudi Arabia; 2 Department of Medicine and Surgery, College of Medicine, Qassim University, Qassim 52571, Saudi Arabiaalnuwaiserali5@gmail.com (A.A.N.); 3 Department of Medicine and Surgery, College of Medicine, Umm Al-Qura University, Makkah 24268, Saudi Arabia; mushalalotibi@gmail.com (M.A.); sroralgethami@gmail.com (S.A.); 4 College of medicine, King Saud bin Abdulaziz University, Jeddah 22384, Saudi Arabia; 5 Department of Physiology, Collage of Medicine, Imam Abdulrahman bin Faisal University, Dammam 31441, Saudi Arabia

**Keywords:** pulmonary hypertension, inspiratory muscle training, respiratory muscle strength, exercise capacity

## Abstract

**Highlights:**

**What are the main findings?**

- IMT significantly improves inspiratory and expiratory muscle strength and consistently reduces dyspnea and fatigue, with improvements in quality-of-life domains.
- IMT is safe and well tolerated, effects on spirometry are negligible, and improvements in exercise capacity (6-MWD) show positive trends but do not reach pooled statistical significance.

**What are the implications of the main findings?**

- IMT is a low-burden, safe, and practical intervention that can be readily integrated into routine management to address key functional limitations associated with PH.
- IMT serves as an effective non-pharmacological adjunct to standard medical therapy, enhancing patient management without introducing additional systemic side effects or safety concerns.
- For physiotherapists and rehabilitation specialists, IMT provides an accessible and versatile modality suitable for both supervised clinical programs and home-based rehabilitation.
- IMT can be delivered using portable threshold devices, facilitating wide adoption across diverse care settings and improving patient adherence.

**Abstract:**

(1) Background: Pulmonary hypertension (PH) is characterized by respiratory muscle weakness, limited exercise tolerance, and reduced quality of life, but inspiratory muscle training (IMT) has emerged as a potential non-pharmacological strategy to improve functional outcomes in this population. This systematic review and meta-analysis evaluated the effects of isolated IMT on respiratory function, exercise capacity, symptom burden, and safety in adults with PH. (2) Methods: A systematic search was conducted in accordance with PRISMA guidelines. Randomized controlled trials involving adults with PH who underwent isolated IMT were included, and respiratory muscle strength, spirometric parameters, exercise capacity, dyspnea, fatigue, quality of life, and adverse events were the outcomes that were assessed. Data were pooled using meta-analytic techniques where appropriate. (3) Results: A total of 130 participants, assigned to five randomized controlled trials, met the inclusion criteria. IMT significantly improved maximal inspiratory pressure (MD = +24.01 cmH_2_O), maximal expiratory pressure (MD = +23.64 cmH_2_O), and six-minute walk distance (MD = +60.61 m), but no significant changes were observed in spirometric indices (FEV_1_%, FVC%, and FEV_1_/FVC). While several individual studies demonstrated clinically relevant improvements in six-minute walk distance, the pooled analysis did not demonstrate a statistically significant effect. IMT consistently reduced dyspnea and fatigue and improved quality-of-life domains. No serious adverse events were reported, and adherence was high. (4) Conclusions: IMT is a safe and feasible adjunct intervention in PH, providing meaningful improvements in respiratory muscle strength and symptom burden. Further large-scale trials are warranted to confirm its long-term clinical benefits.

## 1. Introduction

Pulmonary hypertension (PH) is defined as a mean pulmonary arterial pressure (mPAP) ≥ 25 mmHg [1]. This disease can cause progressive dyspnea, with a variable degree of fatigue and exercise limitation [1,2]. At the global level, PH is found in approximately 1% of elderly individuals, and this rate can increase up to 10% [3,4]. In 2021, a study of the global burden of PH found a prevalence of 2·28 cases per 100,000 population in 204 countries and territories since 1990 [5].

Overall, with this disease, patients’ health-related quality of life (HRQL) decreases, and morbidity and mortality increase [2,6]. According to Miyamoto et al., patients with PH experience symptoms and adverse drug reactions for which there are no established therapies or cures [5]. In addition, patients with PH frequently suffer from psychological disorders, including depression and anxiety, with an increased incidence in more severe cases [7].

Multiple studies evaluated the functional exercise capacity through the six-minute walk distance (6-MWD) test and found that PH severity directly affects performance [8,9,10]. The 6-MWD serves as a vital prognostic indicator for primary PH patients because it shows a strong correlation with mortality risk compared to multiple non-invasive clinical, echocardiographic, and neurohumoral parameters. The survival rate of PH patients decreases as their walking distance shortens [11].

Pharmacological treatments—such as phosphodiesterase-5 inhibitors, prostacyclin, and endothelin-receptor antagonists, which have been used to enhance patients’ hemodynamics and exercise capacity—remain the primary focus of PH management [11]. However, nowadays, healthcare professionals are increasingly interested in accessible and cost-effective non-pharmacological interventions, such as exercise-based programs.

Respiratory muscle training (RMT), particularly IMT, has gained increasing attention as an effective non-pharmacological strategy for improving health status and quality of life in the general population. Evidence from healthy adults and physically active individuals indicates that RMT enhances inspiratory muscle strength and endurance, leading to improved ventilatory efficiency and reduced respiratory effort during daily activities and exercise. These physiological adaptations are associated with lower perceptions of breathlessness, delayed onset of respiratory muscle fatigue, and improved tolerance to physical exertion, all of which positively contribute to perceived well-being and functional independence [12].

Importantly, improvements in respiratory efficiency may also reduce overall cardiorespiratory strain, thereby supporting sustained engagement in physical activity, a key determinant of long-term health and quality of life. Furthermore, RMT has been shown to yield psychosocial benefits, including improved self-efficacy, reduced fatigue, and enhanced health-related quality-of-life scores, even in non-clinical populations. Given its simplicity, low cost, and suitability for home-based implementation, RMT represents a scalable and accessible intervention that can promote respiratory health and overall quality of life in the general population [13,14].

Recent research has shown that supervised exercise-based therapies improve the exercise capacity of PH patients while producing fewer severe side effects than medications and leading to substantial HRQL improvements [15]. For instance, home-based IMT provides a practical solution for patients who cannot participate in structured exercise programs, as it is effective across different disease severities and comorbidities, as well as for those with limited access to rehabilitation centers. IMT enhances respiratory muscle strength, which leads to better ventilatory efficiency, reduced dyspnea, and improved exercise capacity in different cardiorespiratory conditions [16].

Although IMT has gained attention as an adjunct therapy for PH [17], evidence remains fragmented, and no comprehensive quantitative synthesis has yet established its overall effects on clinically meaningful outcomes.

The lack of a comprehensive systematic review and meta-analysis of randomized controlled trials hampers the ability to draw definitive conclusions, limits translation into clinical practice, and restricts the development of evidence-based rehabilitation recommendations. A rigorous synthesis of the available evidence is therefore warranted to clarify the effectiveness of IMT, explore sources of heterogeneity, and guide future research directions in PH. Accordingly, this study was designed to conduct a systematic review and meta-analysis of randomized controlled trials to critically assess the efficacy of inspiratory muscle training in the targeted patient population.

## 2. Materials and Methods

This systematic review and meta-analysis were performed in compliance with the Preferred Reporting Items for Systematic Reviews and Meta-Analyses (PRISMA 2020) reporting standards [2], and the protocol was registered in PROSPERO (CRD420251119297). The eligibility criteria were developed using the Patient, Intervention, Comparison, Outcome (PICO) framework, which defines the population, intervention, comparator, and outcomes (Table 1).

The methodological quality and risk of bias of included randomized controlled trials were assessed using standardized criteria, focusing on random sequence generation, allocation concealment, blinding of outcome assessment, and completeness of outcome data. The overall study quality was low to moderate, with limitations primarily related to blinding and small sample sizes.

For the meta-analysis, effect sizes were calculated as mean differences (MDs) with 95% confidence intervals for continuous outcomes, as all included studies assessed outcomes using identical measurement units. Statistical heterogeneity was assessed using the I^2^ statistic, and random-effects models were applied to account for between-study variability.

### 2.1. Eligibility Criteria

Eligible studies included adult participants (≥18 years) with a confirmed diagnosis of PH, regardless of subgroup classification, who underwent isolated IMT delivered through standardized protocols (typically 30–60% of maximal inspiratory pressure) for a minimum duration of six weeks. Comparator groups consisted of sham IMT or standard care without training.

Reported outcomes included at least one clinically relevant measure from these key areas: pulmonary function [maximal inspiratory pressure (MIP); maximal expiratory pressure (MEP); forced expiratory volume in 1 s (FEV_1_%); forced vital capacity (FVC%); and ratio of forced expiratory volume in 1 s to forced vital capacity (FEV_1_/FVC)], exercise performance indices [6-MWD and peak oxygen uptake (peak VO_2_)], symptom burden [dyspnea or fatigue perception], quality of life, or adverse events. Only randomized controlled trials available in English were included.

Studies were excluded if they involved pediatric populations, if patients had other chronic respiratory conditions (COPD or asthma) or post-surgical PH without a baseline diagnosis, or if IMT was combined with other exercise or rehabilitation modalities. Non-randomized designs, observational studies, case reports, protocols, abstracts without full data, and duplicate publications were excluded. In addition, trials that failed to provide quantitative data on pre- and post-intervention outcomes or demonstrated a high risk of bias and poor methodological quality were not considered.

### 2.2. Search Strategy

Data for this study were collected from PubMed, Google Scholar, ScienceDirect, EMBASE, and CENTRAL using keywords such as (“Pulmonary Hypertension” OR “pulmonary arterial hypertension” OR “PH”) AND (“Inspiratory muscle training” OR “IMT” OR “respiratory muscle training” OR “inspiratory threshold loading”).

### 2.3. Screening Process and Data Extraction

Two reviewers (AZ and SS) independently screened the titles and abstracts of all identified studies using the Rayyan web and mobile application for systematic reviews [18]. The full texts of articles deemed potentially eligible were independently evaluated by two additional reviewers (SN and LA), with any discrepancies resolved through discussion and consultation with a third reviewer (MS). Subsequent data extraction was independently performed by two reviewers (IA and MS), focusing on the following variables: (1) name of the journal, (2) year of publication, (3) last name of the first author, (4) sample size, (5) country of study, (6) study design, (7) mean age of participants, (8) age range of participants, (9) number of female participants, (10) number of male participants, (11) PH classification, (12) WHO functional class, (13) intervention duration and intensity, (14) MIP at baseline and post-training, (15) MEP, (16) FEV_1_%, (17) FVC%, (18) FEV_1_/FVC%, (19) 6-MWD, (20) dyspnea scale, (21) fatigue scale, (22) adverse events, (23) training adherence, and (24) Quality of Life (QoL) tools. All extracted data were double-checked by AZ and SS to ensure accuracy and confirm that no duplication was present.

### 2.4. Risk of Bias Assessment

Risk of bias was independently evaluated by two reviewers using the Revised Cochrane Risk of Bias tool for randomized trials (RoB 2). The assessment covered five methodological domains: bias related to the randomization process, deviations from the intended interventions, missing outcome data, outcome measurement, and selective reporting of results. Each domain was classified as presenting a low risk of bias, some concerns, or a high risk of bias, and an overall judgment was determined in accordance with the Cochrane RoB 2 guidance. Any discrepancies between reviewers were resolved through discussion and consensus.

## 3. Results

**Figure 1:**
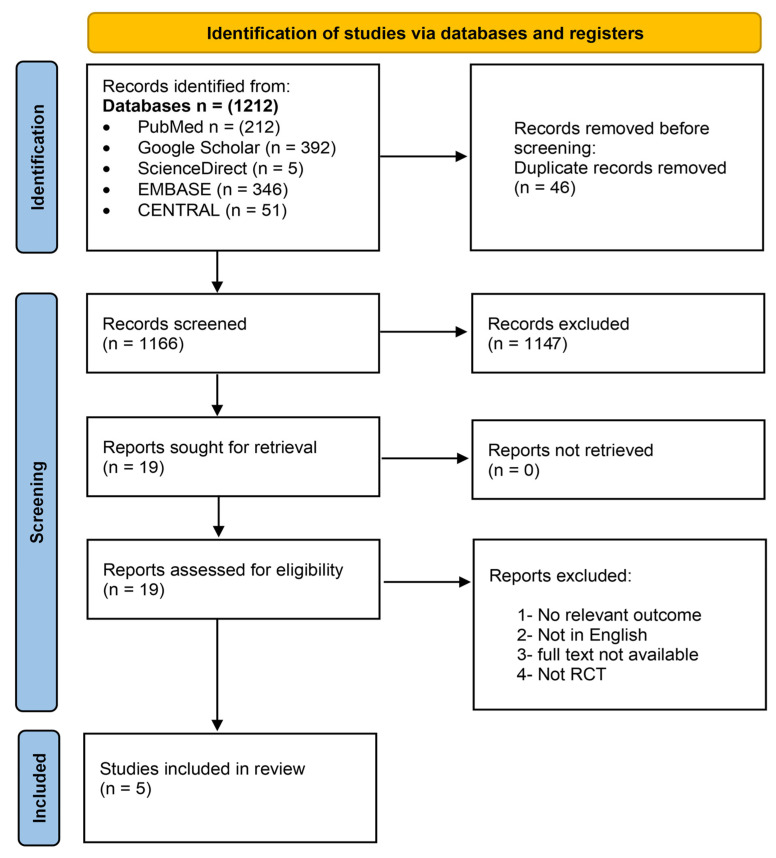
PRISMA flowchart.

A total of 1212 records were retrieved from database searches. After the removal of 46 duplicate entries, 1166 unique records were screened. Based on the evaluation of titles and abstracts, 1148 records were excluded and 18 articles proceeded to the full-text review stage. Of these, five studies fulfilled the eligibility criteria and were ultimately included in the review (Figure 1).

### 3.1. Characteristics of the Included Studies

The present systematic review included a total of 130 participants diagnosed with pulmonary hypertension, with approximately 65 allocated to inspiratory muscle training (IMT) and 65 to control conditions. The five included randomized controlled trials were conducted in Turkey (n = 3), Australia (n = 1), and Brazil (n = 1) and were published between 2015 and 2025. Sample sizes ranged from 12 to 35 participants, with reported mean ages varying approximately from 39 to 60 years. Across all studies, the populations were predominantly female.

Idiopathic pulmonary hypertension represented the most common diagnosis across the included trials, with additional cases of chronic thromboembolic pulmonary hypertension (CTEPH) and connective tissue disease-associated PH also being reported (Table 2). In terms of baseline World Health Organization (WHO) functional classes, severity was primarily categorized as classes II and III.

Interventions consisted of isolated IMT delivered using threshold loading or POWER breathe devices, with training intensities typically ranging from 30% to 60% of maximal inspiratory pressure over 6 to 8 weeks. Control groups received either sham IMT at low intensity or usual care without inspiratory muscle training (Table 2).

### 3.2. Maximal Inspiratory Pressure (MIP)

**Figure 2:**
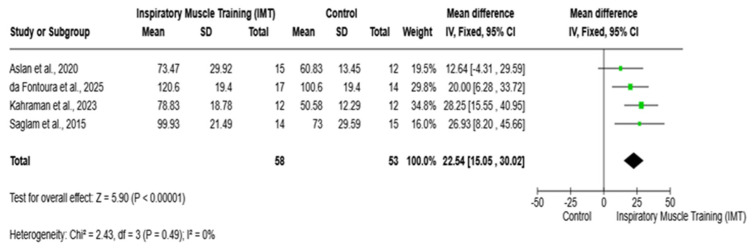
Meta-analysis of maximal inspiratory pressure following inspiratory muscle training. IMT: inspiratory muscle training; MIP: maximal inspiratory pressure; CI: confidence interval; MD: mean difference. Saglam et al., 2015 [8]; Aslan et al., 2020 [19]; da Fontoura et al., 2025 [20]; Kahraman et al., 2023 [21].

Four studies involving 111 participants (IMT: n = 58; control: n = 53) were included in the meta-analysis [8,19,20,21]. Inspiratory muscle training (IMT) resulted in a significant improvement in maximal inspiratory pressure compared with the control, with a pooled mean difference of 22.54 cmH_2_O (95% CI 15.05–30.02; Z = 5.90, *p* < 0.00001). All included studies favored IMT, with mean differences ranging from 12.64 to 28.25 cmH_2_O. No heterogeneity was detected (τ^2^ = 0.00; χ^2^ = 2.43; df = 3; *p* = 0.49; I^2^ = 0%), indicating a consistent and robust effect across studies (Figure 2).

### 3.3. Maximal Expiratory Pressure (MEP)

**Figure 3:**
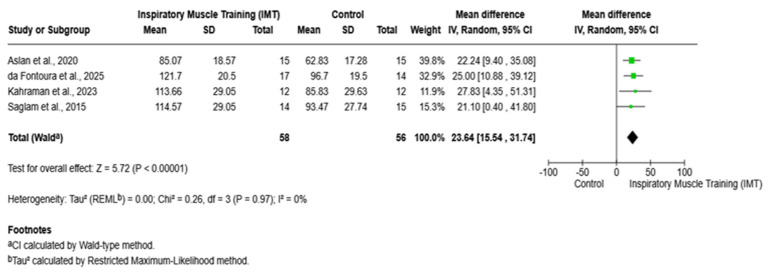
Pooled effect of inspiratory muscle training on maximal expiratory pressure. IMT: inspiratory muscle training; MIP: maximal inspiratory pressure; CI: confidence interval; MD: mean difference. Saglam et al., 2015 [8]; Aslan et al., 2020 [19]; da Fontoura et al., 2025 [20]; Kahraman et al., 2023 [21].

Four randomized controlled trials reported post-training MEP. Pooled results demonstrated a significant improvement in MEP following inspiratory muscle training compared with the control (mean difference = 23.64 cmH_2_O; 95% CI 15.54 to 31.74; Z = 5.72; *p* < 0.00001; I^2^ = 0%) (Figure 3).

### 3.4. Forced Expiratory Volume in 1 Second (FEV_1_%) and Forced Vital Capacity (FV%)

**Figure 4:**
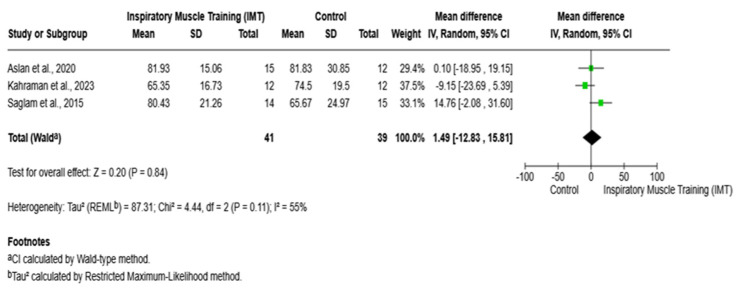
Forest plot of the effect of IMT versus the control on FEV_1_%. IMT: inspiratory muscle training; FEV_1_%: forced expiratory volume in 1 s; CI: confidence interval; MD = mean difference. Saglam et al., 2015 [8]; Aslan et al., 2020 [19]; Kahraman et al., 2023 [21].

The pooled analysis of three randomized controlled trials [8,19,21] evaluating the effect of IMT on FEV_1_% demonstrated no significant difference between the IMT and control groups. The overall mean difference was 1.49% (95% CI: −12.83 to 15.81; *p* = 0.84) (Figure 4).

**Figure 5:**
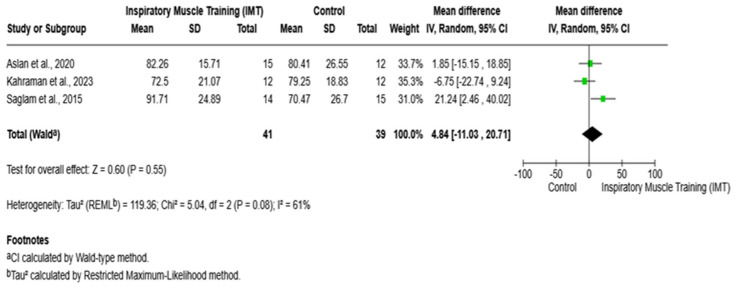
Forest plot of the effect of IMT versus control on FVC%. IMT: inspiratory muscle training; FEV_1_%: forced expiratory volume in 1 s; CI: confidence interval; MD = mean difference. Saglam et al., 2015 [8]; Aslan et al., 2020 [19]; Kahraman et al., 2023 [21].

Regarding forced vital capacity (FVC% predicted), the mean differences ranged from −6.75 to 21.24. Similarly, the pooled results derived from a random-effects model demonstrated no statistically significant difference in forced vital capacity (FVC%) between the inspiratory muscle training (IMT) and control groups (mean difference = 4.84; 95% confidence interval: −11.03 to 20.71; *p* = 0.55) as presented in Figure 5.

**Figure 6:**
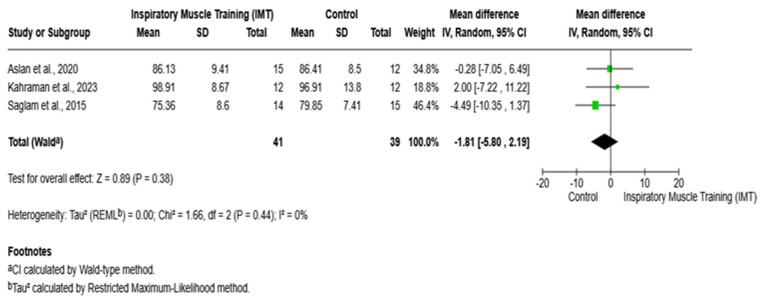
Forest plot of the effect of IMT versus control on FEV_1_/FVC (%). IMT: inspiratory muscle training; FEV_1_%: forced expiratory volume in 1 s; FVC: forced vital capacity; CI: confidence interval; MD = mean difference. Saglam et al., 2015 [8]; Aslan et al., 2020 [19]; Kahraman et al., 2023 [21].

With respect to the FEV_1_/FVC ratio (%), the findings indicated no statistically significant differences between the IMT and control groups. Across the included studies, mean differences ranged from −4.49 to 2.00, and a pooled analysis demonstrated that IMT was not associated with a significant improvement in FEV_1_/FVC compared with the control (mean difference = −1.81; 95% confidence interval: −5.80 to 2.19; *p* = 0.38), as illustrated in Figure 6.

### 3.5. Six-Minute Walk Distance (6-MWD, Meters)

**Figure 7:**
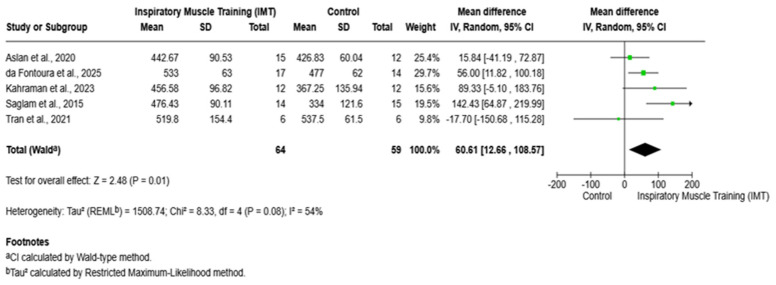
Forest plot of the effect of IMT versus the control on the 6-MWD test. IMT: inspiratory muscle training; 6-MWD: six-minute walk distance; CI: confidence interval; MD: mean difference. Saglam et al., 2015 [8]; Tran et al., 2021 [16]; Aslan et al., 2020 [19]; da Fontoura et al., 2025 [20]; Kahraman et al., 2023 [21].

Five studies, with 123 participants (IMT: n = 64; control: n = 59), were included in the meta-analysis [8,16,19,20,21]. IMT was associated with a significant improvement in the 6 min walk distance test compared with the control, with a pooled mean difference of 60.61 m (95% CI 12.66–108.57; Z = 2.48; p = 0.01). Most studies favored IMT, although one study reported a non-significant effect. Moderate heterogeneity was observed among studies (p = 0.08; I^2^ = 54%), suggesting some variability in effect size across trials (Figure 7).

Moreover, previous studies havereported improvements in exercise capacity following inspiratory muscle training (IMT), as reflected by increases in the 6-MWD ranging from approximately 24 to 51 m [8,16,19,20,21]. Reductions in dyspnea and fatigue were observed in several studies, with statistically significant improvements reported by Kahraman et al. (2023) [21] and Saglam et al. (2015) [8], as well as by da Fontoura et al. (2025) [20] using Borg and mMRC scales. Studies assessing health-related outcomes demonstrated improvements in quality of life, which was measured using the MLHFQ (Aslan et al., 2020) [19], SF-36 (da Fontoura et al., 2025) [20], and Nottingham Health Profile (Kahraman et al., 2023, and Saglam et al., 2015) [8,21]. In addition, training adherence was high, exceeding 75–90%, and IMT was well tolerated, with no serious adverse events reported; only mild, transient muscle discomfort was noted in a small number of participants [8,16,19,20,21] (Table 3).

### 3.6. Risk of Bias Assessment (ROB)

**Figure 8:**
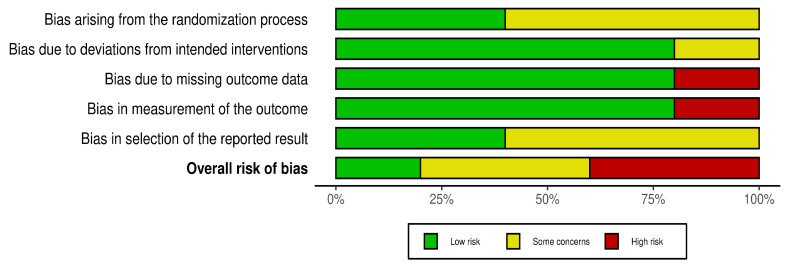
Summary of the risk of bias.

**Figure 9:**
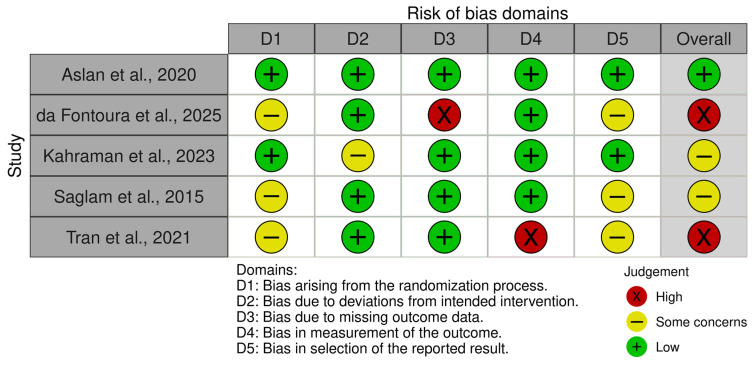
Traffic light plot for the risk of bias. Saglam et al., 2015 [8]; Tran et al., 2021 [16]; Aslan et al., 2020 [19]; da Fontoura et al., 2025 [20]; Kahraman et al., 2023 [21].

The ROB demonstrated variability across the included studies. One study [19] had an overall low risk of bias across all domains, while three studies [8,16,20] presented some concerns, primarily related to randomization procedures, selection of reported results, or missing outcome data. Among these, studies by da Fontoura et al. (2025) [20] and Tran et al. (2021) [16] were rated as having a high overall risk of bias due to issues with missing outcome data and selective reporting, and there were some concerns with Kahraman et al. (2023)’s study [21], particularly in the domain of randomization (Figure 8 and Figure 9).

## 4. Discussion

In this systematic review and meta-analysis, IMT was shown to confer meaningful benefits in adults with PH, particularly with respect to respiratory muscle performance and symptom burden. Significant improvements were observed in both inspiratory and expiratory muscle strength, suggesting that IMT effectively targets peripheral respiratory muscle dysfunction, which is a recognized contributor to exercise intolerance in PH. In contrast, traditional spirometric measures, including FEV_1_% and FVC%, remained largely unchanged, indicating that the observed functional gains are unlikely to be mediated by alterations in pulmonary mechanics.

Notably, IMT was associated with clinically relevant improvements in functional exercise capacity, alongside reductions in dyspnea and fatigue, underscoring its potential role as a complementary, non-pharmacological intervention aimed at improving patient-centered outcomes in this population.

In our meta-analysis, MIP increased by 24 cmH_2_O and MEP by 24 cmH_2_O (both *p* < 0.00001). These findings are consistent with a study by Laohachai et al. (2017) [22], which demonstrated that a six-week IMT program resulted in significant improvements in inspiratory muscle strength and enhanced ventilatory efficiency during exercise. Such adaptations align with established physiological principles of respiratory muscle conditioning. Moreover, according to the statement from the American Thoracic Society/European Respiratory Society (ATS/ERS) on respiratory muscle testing, a maximal inspiratory pressure (MIP) threshold of approximately 80 cmH_2_O is considered necessary to meaningfully address inspiratory muscle weakness [23]. Collectively, these observations support the premise that sufficiently dosed IMT can elicit clinically relevant respiratory muscle adaptations, which may contribute to improved exercise performance in individuals with compromised cardiopulmonary function.

In this review, higher-intensity protocols showed greater gains. For instance, Kahraman et al. (40–60% MIP) documented robust strength gains and even increases in diaphragm thickness [21]. In contrast, lower-load training, such as in Aslan et al. (2020)’s study at 30% MIP, produced lower MIP gains [19].

In contrast to muscle strength, IMT had no impact on spirometric lung volumes. Our pooled analysis showed no significant changes in FEV_1_% predicted, FVC% predicted, or the FEV_1_/FVC ratio with IMT (*p* > 0.05). This is concordant with existing evidence in 2025, which showed effects of IMT in PH patients, with no changes in FEV_1_ and FVC [24]. FEV_1_ and FVC are force-dependent measurements that reflect lung capacity and are primarily affected by respiratory muscle strength, airway resistance, and lung compliance [25].

Despite significant improvements in respiratory muscle strength, these gains did not translate into measurable changes in static spirometric lung volumes because IMT targets the respiratory musculature rather than the lung parenchyma. As a result, improvements in muscle force are not expected to translate into large changes in static lung volumes or flows [26].

Moreover, the pooled analysis showed a statistically significant improvement in 6-MWD. However, moderate heterogeneity and small sample sizes may reduce confidence in the precision of this estimate. Our meta-analysis showed a 60.61 m increase in 6-MWD with IMT (95% CI 12.66–108.57, *p* = 0.01). This trend is in line with an evidence review that highlighted that the 39-m improvement in 6-MWD is close to the minimal clinically important difference reported for chronic respiratory diseases [24].

Mechanistically, enhanced inspiratory muscle strength is expected to reduce the work of breathing and strain on the respiratory muscle, thereby preserving leg muscle blood flow and delaying fatigue during walking [27]. Moreover, IMT appeared to reduce symptom burden and improve quality of life, although results were variable. After IMT, Saglam et al. (2015) observed reduced dyspnea (mMRC) and fatigue (FSS) scores, along with better emotional status on the Nottingham profile [9].

Kahraman et al. (2023) similarly found that IMT significantly lowered dyspnea and fatigue impact while enhancing daily activity and several QoL dimensions [21]. Meanwhile, Aslan et al. (2020) found no between-group differences in dyspnea, fatigue, or Minnesota Living with Heart Failure QoL scores [19].

Importantly, the consistent direction of symptom relief across studies suggests a true physiological effect. Another study found that QoL score improved in the IMT group (*p* = 0.03) [28]. Variable QoL outcomes in research stem from methodological factors such as small samples, questionnaire sensitivity, and baseline. However, the positive trends in physical and emotional domains support a beneficial role for IMT [29].

The findings of this systematic review and meta-analysis have direct implications for clinical practice across multidisciplinary PH care teams. IMT may offer physicians a safe adjunct to standard therapy and provides physiotherapists and rehabilitation specialists with an accessible rehabilitation modality that enhances respiratory muscle strength, exercise tolerance, and QoL in PH.

### Limitations and Recommendations

Several limitations were noted across the included studies. These primarily involve small sample sizes and the clinical heterogeneity of PH patients, which limit the generalizability of the findings. The use of generic QoL questionnaires, rather than disease-specific tools, was also identified as a limitation in some studies, potentially contributing to the underestimation of the true impact on patients’ well-being. Future studies with larger, multicenter RCTs with longer follow-up durations, utilizing disease-specific QoL measures and exploring optimal IMT intensities and durations, are needed. Moreover, comparative studies between isolated IMT and combined exercise programs, as well as investigations into the underlying physiological mechanisms through more objective measures, are also warranted.

## 5. Conclusions

The present systematic review and meta-analysis suggest that IMT is a safe, feasible, and potentially clinically beneficial intervention for patients with PH, with observed improvements in respiratory muscle strength, exercise tolerance, and symptom perception. Reductions in dyspnea and fatigue, alongside gains in selected QoL domains, indicate that IMT may help address key functional limitations in this population. However, these findings should be interpreted with caution, as the overall certainty of evidence is influenced by methodological limitations within the included trials, including risks related to blinding, sample size, and between-study heterogeneity. IMT appears particularly relevant for patients with marked inspiratory muscle weakness and advanced exercise intolerance, but confirmation of its clinical effectiveness requires further high-quality, adequately powered randomized trials. Future research should prioritize rigorous study designs, standardized outcome measures, and optimized IMT prescription parameters and evaluate its integration within comprehensive pulmonary rehabilitation models to support long-term clinical outcomes.

## Figures and Tables

**Table 1:** PICOS criteria for inclusion and exclusion of studies.

PICO Element	Description
Population (P)	Adults (≥18 years) diagnosed with pulmonary hypertension (any classification group) based on established clinical criteria.
Intervention (I)	Isolated IMT delivered through standardized protocols (30–60% of maximal inspiratory pressure), with a minimum duration of six weeks.
Comparator (C)	Sham IMT or usual care without inspiratory muscle training.
Outcomes (O)	Pulmonary function parameters (MIP, MEP, FEV_1_%, FVC%, and FEV_1_/FVC), exercise capacity (6-MWD and peak VO_2_), symptom burden (dyspnea/fatigue perception), quality of life, and adverse events.

IMT: inspiratory muscle training; MIP: maximal inspiratory pressure; MEP: maximal expiratory pressure; FEV_1_%: forced expiratory volume in 1 s; FVC%: forced vital capacity; FEV_1_/FVC: ratio of forced expiratory volume in 1 s to forced vital capacity; 6-MWD: six-minute walk distance; Peak VO_2_: peak oxygen uptake.

**Table 2:** Characteristics of the included studies.

Study ID (Last Name, Year)	Country	Study Design	Sample Size	Age Year (Mean ± SD/Range)	M	F	PH Classification	WHO Functional Class	Intervention: Duration and Intensity
Aslan, 2020 [19]	Turkey	RCT, single-blinded	30	48 ± 10/30–70	4	23	Idiopathic 13 (48.1%); CTD-PH 7 (25.9%); CTEPH 7 (25.9%)	I 8 (29.6%); II 12 (44.4%); III 7 (25.9%)	8 weeks; 15 min × 2/day, 5 days/week; TIMT 30% MIP, Sham 9 cm H_2_O
da Fontoura, 2025 [20]	Brazil	RCT, single-blinded	35	38.9 ± 8.5/NR	0	35	PH 29; CTEPH 2	II 71%, III 29%	8 weeks; 50% PImax, twice daily
Kahraman, 2023 [21]	Turkey	Randomized controlled, evaluator-blinded trial	24 (IMT = 12, Control = 12)	IMT 49.16 ± 17.09; Control 55.50 ± 19.17/NR	2	22	Idiopathic PH (58.3%); PH-CHD (IMT 25%, control 16.7%); PH-CTD (control 16.7%); CTEPH (IMT 16.7%, control 8.3%)	II (IMT 75%, control 58.3%); III (IMT 25%, control 41.7%)	40–60% MIP, adjusted weekly; 30 min/day, 7 days/week (6 home, 1 supervised), 8 weeks
Sağlam, 2015 [8]	Turkey	Prospective randomized controlled trial	29 (IMT = 14, Control = 15)	49.7 ± 12.4/NR	6	25	PH	II (51.6%), III (48.4%)	IMT with inspiratory threshold device, 30% MIP, 30 min/day, 7 days/week, 6 weeks. Control: sham IMT at 10% MIP
Tran, 2021 [16]	Australia	Pilot randomized controlled trial	12 (IMT = 6, Control = 6)	60 ± 14/NR	2	10	IMT: PH 5, CTEPH 1; Control: PH 5, CTEPH 1	IMT: II 5, III 1; Control: II 6, III 0	IMT 30–40% PImax, POWERbreathe KHP2, 2 × 30 breaths/day, 5 days/week, 8 weeks. Control: routine management only

M: males; F: females; NR: not reported; PH: pulmonary hypertension; CTEPH: chronic thromboembolic pulmonary hypertension; WHO Functional Class: World Health Organization functional class; IMT: inspiratory muscle training; MIP: maximal inspiratory pressure; SD: standard deviation. Sample size refers to the number of randomized participants. Training intensity is expressed as a percentage of baseline MIP unless otherwise noted.

**Table 3:** IMT effects on exercise capacity, quality of life, adherence, and safety in patients with PH.

Study (Year, Country)	Exercise Capacity (6-MWD, Dyspnea, Fatigue)	Quality of Life	Training Adherence	Adverse Events
Aslan 2020, Turkey [19]	6-MWD ↑ ~24 m, dyspnea and fatigue: no significant change	MLHFQ: improved	100% completed	None reported
da Fontoura 2025, Brazil [20]	6-MWD ↑ +33 m, Borg and mMRC ↓, fatigue ↓ slightly	SF-36v2: improved	IMT 77%, sham 83% sessions completed	Mild muscle pain in 5 pts, no dropouts
Kahraman 2023, Turkey [21]	6-MWD ↑ +51 m (*p* < 0.001), Borg dyspnea ↓ (*p* < 0.001), fatigue ↓ (*p* = 0.003)	Nottingham Health Profile: improved	Good; 8-week program (home + supervised)	None
Sağlam 2015, Turkey [8]	6-MWD ↑ +49 m (*p* < 0.05), dyspnea ↓, fatigue ↓	Nottingham Health Profile: improved	High adherence, no dropouts	1 mild muscle soreness only
Tran 2021, Australia [16]	6-MWD ↑ +36 m (*p* = 0.03), dyspnea/fatigue not reported	Not assessed	98% cycles completed	None

↑: significant increase; ↓: significant decrease; 6-MWD: six-minute walk distance; MLHFQ: Minnesota Living with Heart Failure questionnaire.

## Data Availability

No new data were generated or analyzed in this systematic review. All data supporting the findings of this study are derived from previously published articles, which are cited in the reference list.
